# Simpson-Golabi-Behmel-Syndrome in Dichorionic-Diamniotic Twin Pregnancy

**DOI:** 10.3390/clinpract11010012

**Published:** 2021-02-02

**Authors:** Theresa Reischer, Franco Laccone, Gregor J. Kasprian, Gülen Yerlikaya-Schatten

**Affiliations:** 1 Department of Obstetrics and Feto-maternal Medicine, Medical University of Vienna, 1090 Vienna, Austria; theresa.reischer@meduniwien.ac.at; 2 Institute of Medical Genetics, Medical University of Vienna, 1090 Vienna, Austria; franco.laccone@meduniwien.ac.at; 3 Department of Radiology, Division of Neuro- and Musculoskeletal Radiology, Medical University of Vienna, 1090 Vienna, Austria; gregor.kasprian@meduniwien.ac.at

**Keywords:** prenatal diagnosis, exome sequencing, twin pregnancy, Simpson-Golabi-Behmel-syndrome

## Abstract

Simpson-Golabi-Behmel syndrome (SGBS) is a rare x-linked overgrowth syndrome with distinct clinical features, which is difficult to diagnose prenatally. We report the diagnosis of SGBS in dichorionic-diamniotic twin pregnancies in the first trimester by ultrasound and genetic testing. The affected fetus developed polyhydramnios and the cervical length of the mother decreased significantly. To save the unaffected twin, a selective feticide of the affected fetus was performed. Finally, the patient underwent preterm caesarean section due to premature rupture of membranes in the dead twin, and also intrauterine infection. While SGBS has been reported, this was the first case in a multiple pregnancy, with possible consequences for the healthy twin. In conclusion, SGBS is a rare condition, which should be considered in the differential diagnosis of prenatal overgrowth syndromes and associated malformation.

## 1. Introduction

Simpson-Golabi-Behmel syndrome counts as overgrowth syndrome. These are a heterogeneous group of disorders characterized by enormous growth of the affected individual. The Simpson-Golabi-Behmel syndrome (SGBS) is an X-linked recessive disorder. It was first described by Simpson in 1975 [1]. Subsequently, Behmel et al., Golabi, and Rosen reported several affected men with similar symptoms [2,3]. SGBS is characterized by massive pre- and postnatal overgrowth, a disproportionately large head, variable congenital malformations such as hyperteleorisim, renal abnormalities, cardiac, gastrointestinal and genitourinary malformations, as well as embryonal tumours and increased mild to moderate intellectual shortfall [3,4]. The syndrome is caused by pathogenic variants in the glypican-3 gene (*GPCFF3*-Gene) on chromosome X. It is inherited in an X-chromosomal recessive manner [5]. The vast majority of variants are frameshift or nonsense variants resulting in loss of function [6]. There are two types of SGBS: Type 1 and 2. SGBS Type 1 (OMIM 312870) is a milder form in comparison to SGBS Type 2 (OMIM 300209), which is associated with hydrops fetalis and multiple anomalies [7]. Female carriers are most likely asymptomatic, whereas all male individuals are affected. 

With the advances in prenatal diagnosis within the last years, diagnosis of SGBS has been described but only in single cases. This is because prenatal features of SGBS type 1 can vary widely in affected individuals. Ultrasound findings may include increased nuchal translucency (NT) measurements, renal anomalies, polyhydramnios, and congenital diaphragmatic hernia [7]. In spite of known ultrasound features the prenatal detection rate is low. This case report describes the diagnosis of SGBS in a twin pregnancy with fetal abnormalities detectable by ultrasound as early as 12 weeks of pregnancy.

## 2. Case Report

**Figure 1:**
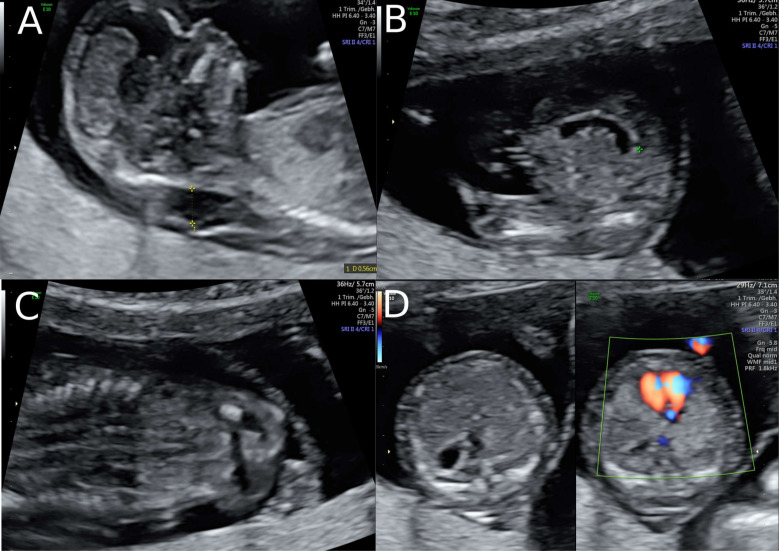
Ultrasound findings in the 1st Trimester. (**A**) Increased Nuchal Translucency (NT) at 13 + 5 weeks of gestation in twin II. (**B**) A simple cystic structure in the abdomen, most probably a gastrointestinal duplication cyst. (**C**) Hyperechogenic kidneys. (**D**) Malposition of the fetal heart in the chest at 13 + 5 weeks. The position of the fetal heart in the middle of the thorax.

**Figure 2:**
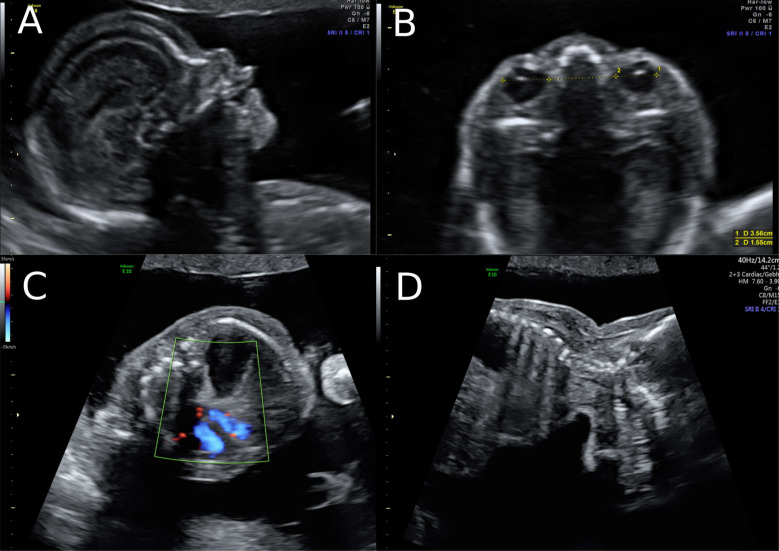
Ultrasound at 22 weeks of gestation (**A**,**B**) Striking profile and brain structure in fetus with Simpson-Golabi-Behmel syndrome. **(C)** Suspicion of congenital diaphragmatic hernia at 22 weeks of gestation and (**D**) increased subcutaneous fat tissue.

**Figure 3:**
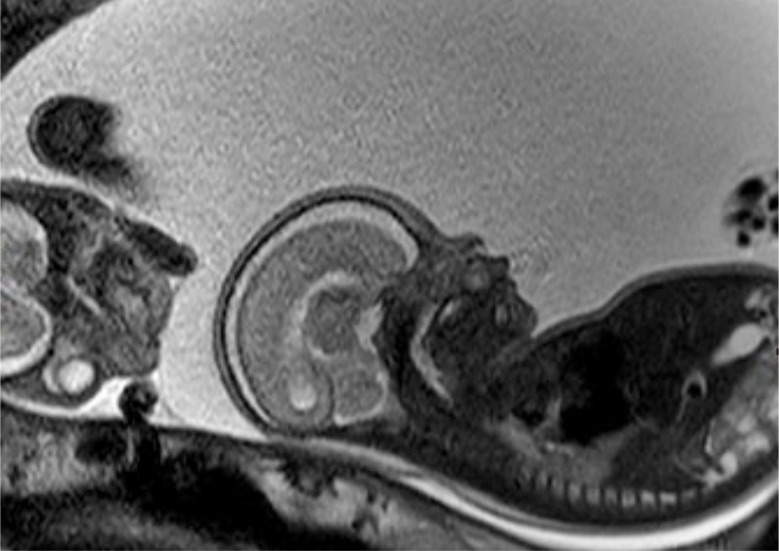
Fetal MRI at 25 weeks of gestation: the fetal MRI picture reveals the massive polyhydramnios. Due to the polyhydramnios of fetus II, fetus I provided a similar picture like a stuck twin, as it is found in a twin-to-twin transfusion syndrome, although fetal development and amniotic fluid have been normal in the second twin.

A 38-year-old women with a dichorionic-diamniotic twin pregnancy, after in-vitro fertilisation, attended routine first trimester screening at 13 + 4 weeks of gestation. One of the twins (fetus I) pair showed normal NT measurement and a low risk for chromosomal abnormalities, whereas the other twin (fetus II) presented an increased NT of 5.2 mm, the position of the fetal heart in the middle of the thorax, and hyperechogenic kidneys (Figure 1). At this stage, the patient declined invasive diagnosis. At 15 + 5 weeks of gestation, additional findings to the ones above, including hydronephrosis, skin oedema, and a right-sided clubfoot, have been observed. Subsequently, amniocentesis in the affected fetus was performed and revealed a 46, XY karyotype. Estimated fetal weight for both were average for the gestational age but abdominal circumference (AC) of the second twin was at the 95th percentile. At 22 + 0 weeks of gestation, the overall estimated fetal weight of fetus II was above the 95th percentile and a nuchal fold of 8.1 mm with general oedema of the skin was present. The ultrasound scan additionally showed a striking profile (Figure 2). All measurements (HC-AC-FL) were above the 95th percentile. A suspicion of left-sided diaphragmatic hernia as well as hyperechogenic kidneys, increased in size and polycystic appearance, and a hypoplasia of genitalia have been identified. Furthermore, the scan revealed a polyhydramnios (DIP 10 cm). Due to the polyhydramnios of fetus II, fetus I has been compromised in a similar process as twin-to-twin transfusion syndrome, although fetal development and amniotic fluid have been normal (Figure 3). 

Whole exome sequencing revealed a hemizygous canonical donor splice site of the *GPC3*-Gene (NM_004484.3: c.175 + 1G > T) which causes SGBS. The Sanger sequencing of the mother could confirm that she is a heterozygous carrier of this mutation. There was no known family history of SGBS. After genetic counseling, the patient underwent fetal MRI to gain additional information as selective feticide has been discussed with the couple. The MRI revealed a conspicuousness of the diaphragm, i.e., diaphragmatic hernia versus eventration, with the stomach and the spleen being mostly positioned intrathoracic, as well as parts of the left and right lobe of the liver. Thus, consecutive moderate lung hypoplasia with a lung volume of 6.8mL on the right and 11 mL on the left side. Bilateral, multicystic kidneys with kidney parenchyma residual on both sides were described. Furthermore, the brain examination revealed accentuated ventricular width within the atrium area with especially frontal irregular ventricular confinement. All ultrasound and MRI findings are listed and compared to previous cases in Table 1. 

After detailed counseling the couple decided to have selective feticide. Due to the massive polyhydramnios and cervix insufficiency the patient was admitted to the hospital firstly for lung maturation with corticosteroid and secondly selective feticide at 25 + 0 weeks of gestation. However, before feticide, amniotic fluid drainage had to be done. Therefore, 2900 mL amniotic fluid had been drained and, subsequently, selective feticide through intracardiac injection was carried out. The patient was discharged the following day. At 25 + 5 weeks of gestation, the patient was admitted to the hospital due to rupture of the membranes in the death twin and caesarean section was performed at 31 + 1 weeks due to signs of intrauterine infection.

## 3. Outcome and Follow-Up

The healthy baby girl was transferred to the intensive care unit with 1700 g and Apgar 7/8/9. The carrier status of the newborn for *GPC3*-Gene is unknown up to this time point, but the phenotype of the newborn girl was normal. The recovery of the mother was uneventful, and she could be discharged after a few days. The newborn was discharged after eight weeks in hospital without any sequelae.

## 4. Discussion Include a Very Brief Review of Similar Published Cases 

This case report describes the first cases of Simpson-Golabi-Behmel syndrome in a twin pregnancy, diagnosed prenatally. 

Prenatal diagnosis of Simpson-Golabi-Behmel syndrome is difficult as the sonographic findings appear mostly after the second trimester and usually are not very specific for SGBS. Nevertheless, there have been reports in the literature that an increased NT measurement could be an early sign for this condition or even overgrowth syndrome in general [7,8]. However, SGBS is a rare condition, and an elevated NT is a non-specific marker occurring in many genetic and non-genetic conditions. Moreover, performing a whole exome sequencing (WES) for isolated increased NT is not recommended due to low diagnostic output published before [9]. Therefore, the clinical implementation of WES should be considered carefully, as the overall detection rate of diagnostic genetic variant depends on additional fetal malformation as well as on the test method [10]. Recent studies indicate a higher diagnostic yield of whole genome sequencing in fetuses with increased nuchal translucency. However, due to the sparse literature available, prospective studies with further evidence and larger sample size have to prove the clinical implementation of whole genome sequencing within this indication [11]. In the present case, WES seemed reasonable as there were multiple malformations and the karyotype was inconspicuous. The sequence analysis detected a variant affecting a canonical splice site of the gene: c.175G > T. The same position has already been described by Vuillaume et al., [6] in a case of SGBS: c.175G > A, which supports the pathogenicity of the detected variant of the present case. 

Prenatal diagnosis of such a severe genetic disorder is crucial for the decision to termination of pregnancy for a majority of parents. Especially in twin pregnancies, if, due to the affected fetus, the second fetus will be put at risk. Therefore, timing of feticide and delivery have to be considered carefully.

## 5. Conclusions

Summarized, prenatal diagnosis of SGBS is difficult as the sonographic findings appear late in pregnancy. However, pathognomonic features can lead to the suspicion of SGBS prenatally and justify prenatal exome sequencing. This case demonstrates the significance of prenatal genetic diagnosis and the interdisciplinary challenges of such a pregnancy. Especially in twin pregnancy, timing of feticide and delivery may be critical to not put the healthy twin at risk.

## Figures and Tables

**Table 1:** Summarized ultrasound and MRI findings in Simpson-Golabi-Behmel syndrome (SGBS) type 1 compared with the cases of Cong et al., [7].

	Our Case		Chong et al		
	First trimester	Second trimester	Case 1	Case 2	Case 3
gestational age	13 + 4	22 + 0	19 + 6	21 + 4	18 + 5
elevated NT	5.2mm	8.1 mm	2.3mm	NA	NA
craniofacial anomaly			X	X	
brain anomaly		accentuated ventricular width			
macrosomia		X			
polyhydramnios		X			
visceromegaly			X		
renal anomaly	hyperechogenic kidneys	Bilateral, multicystic kidneys	X	X	X
CDH		left sided CDH	X	X	X
cardiac anomaly			X		
skeletal anomaly		right-sided clubfoot			X
outcome	TOP selective feticide at 25+0 weeks		TOP 21wks	TOP 22wks	TOP 22wks

## Data Availability

No new data were created or analyzed in this study. Data sharing is not applicable to this article.
